# Multimodal prehabilitation for upper gastrointestinal tumors: effects on postoperative morbidity and mortality – a meta-analysis of randomized controlled trials

**DOI:** 10.3389/pore.2026.1612328

**Published:** 2026-04-28

**Authors:** Csenge Papp, Armand Csontos, Daniel Kehl, Lili Dora Sindler, Andras Vereczkei, Andras Papp

**Affiliations:** 1 Department of Surgery, Clinical Center, University of Pécs, Pécs, Hungary; 2 Faculty of Business and Economics, University of Pécs, Pécs, Hungary

**Keywords:** ERAS protocol, meta-analysis, postoperative complications, prehabilitation, upper gastrointestinal cancers

## Abstract

**Background:**

Patients suffering from upper gastrointestinal (UGI) cancers often present with malnutrition and frailty before undergoing major surgical procedures, which significantly elevates the risk of postoperative complications. Prehabilitation focuses on optimizing a patient’s functional capacity before surgery to improve postoperative outcomes. Our goal was to synthesize the effects of prehabilitation on the postoperative outcomes of UGI cancer patients undergoing major surgical intervention using a systematic review and meta-analysis.

**Methods:**

A comprehensive systematic search was conducted across the PubMed, Embase, Cochrane, and Scopus databases to identify relevant randomized controlled trials (RCTs). Ten RCTs, encompassing data from 878 patients, were included in the analysis. Pooled risk ratios (RRs) were calculated for dichotomous variables (e.g., incidence of complications), and weighted mean differences were calculated for continuous variables using a random-effects model. Study quality was assessed using the RoB2 and GRADE approaches.

**Results:**

The meta-analysis showed a trend toward a lower incidence of minor postoperative complications (Clavien–Dindo Grade I–II) in the prehabilitation group. While the common effect model showed significance, the certainty of evidence remains low to very low for most outcomes, suggesting these results should be interpreted with caution. However, a significant reduction was found in Grade III complications when using a common effect model, although no significant differences were detected in Grade IV complications or mortality. Cardiovascular complications and hospital readmission rates also showed no significant disparity.

**Conclusion:**

The implementation of prehabilitation in UGI cancer patients is safe and shows a positive trend toward reducing minor postoperative complications, thereby enhancing patient comfort and potentially accelerating recovery time. While the certainty of evidence remains low, further high-quality RCTs with larger patient cohorts are warranted, especially to explore the role of multimodal prehabilitation.

## Introduction

It is essential to evaluate the nutritional and physical condition of patients with malignant diseases before surgery, particularly for those with upper gastrointestinal (UGI) cancers. These tumors often lead to swallowing difficulties, which can quickly induce a catabolic state even before the operation [1]. The World Health Organization reported approximately 541,019 new cases of oesophageal cancer and 1,002,136 new cases of stomach cancer globally in 2022 [2]. Upper gastrointestinal tract cancers represent a significant global health issue, ranking as the 6th most common cause of cancer-related deaths worldwide in 2020 [3].

Risk factors for these cancers include smoking, alcohol consumption, poor diet, and infection by specific bacteria or viruses, such as *Helicobacter pylori* and human papillomavirus (HPV). For example, HPV infection is a prognostic factor in oropharyngeal squamous cell carcinomas (SCC), where altered miRNA expression patterns have been identified in both tumor tissues and surrounding mucosa. Preventive strategies include quitting smoking, reducing alcohol intake, adopting a healthy diet, and receiving the HPV vaccine [4, 5].

Recently, there has been dynamic progress in the multidisciplinary treatment of cancer patients, and we expect that the development and implementation of a new protocol system for nutritional therapy will become widespread and significantly contribute to further advancements. Applying an appropriate nutritional protocol can improve tissue repair, support the proper functioning of the immune system, and reduce the risk of complications [6, 7].

Malnutrition is one of the main factors contributing to mortality in these patients [7, 8]. Therefore, it is essential to address overall condition of the patients not only after surgery, but also before it. Prehabilitation represents a frequently neglected but, we believe, vital element of the ERAS (Enhanced Recovery After Surgery) protocol, which can be crucial in the preoperative period, especially for patients with stomach and oesophageal cancers. It is important to note here, the present study does not wish to discuss the entire ERAS protocol as instead, it focuses on the prehabilitation phase. However, in Table 1 we marked those RCTs where the entire ERAS protocol was applied. The goal of prehabilitation is to prepare patients physically and psychologically for a major surgery [9, 10].

**TABLE 1:** Comparison of prehabilitation types, ERAS status, and study intervals.

Author	Prehabilitation	ERAS	Surgery	Sample Size (I/C)	Initiation of prehabilitation
Guinan et al.	Physical	+	Esophagectomy	28/32	≥2 weeks preoperative
Valkenet et al.	Physical	-	Esophagectomy	95/121	≥2 weeks preoperative
Zylstra et al.	Physical	-	Esophagectomy	21/19	Started before neoadjuvant therapy
Kong et al.	Nutritional	-	Gastrectomy	65/62	2 weeks preopereative
Liu et al.	Nutritional	+	Esophagectomy	26/24	1 week preoperative
Loughney et al.	Physical	-	Gastrectomy or esophagectomy	36/35	Started before neoadjuvant therapy
Yamana et al.	Physical	-	Esophagectomy	30/30	≥1 week preoperative
Minnella et al.	Nutritional, physical	+	Gastrectomy or esophagectomy	26/25	Followed ERAS principles
Allen et al.	Nutritional, physical, psychological	-	Gastrectomy or esophagectomy	26/28	15-week preoperative program
Bausys et al.	Nutritional, physical, psychological	-	Gastrectomy	64/64	4 weeks preoperative

The use of prehabilitation is becoming increasingly accepted in contemporary medicine, as evidenced by numerous international publications. Based on these studies, we planned a meta-analysis to summarize the impact of prehabilitation on patients with upper gastrointestinal cancers. The aim of our study is to examine the effects of prehabilitation in upper gastrointestinal cancer patients preparing for major surgical interventions.

## Materials and methods

### Protocol registration

The protocol of the meta-analysis methods was registered in database of Prospero previously in the number of 653422. The acceptance of registration was in 2025.02.24.

### The question of the review

The aim of the meta-analysis is defined in PICO protocol. The included patients (P) underwent upper gastrointestinal surgery because of malignancy. The intervention (I) group underwent physical or nutritional prehabilitation before surgery, while the control (C) group received no preoperative intervention. We examined the outcome of postoperative mortality, morbidity and performance status.

### Search strategy and selection process

**FIGURE 1:**
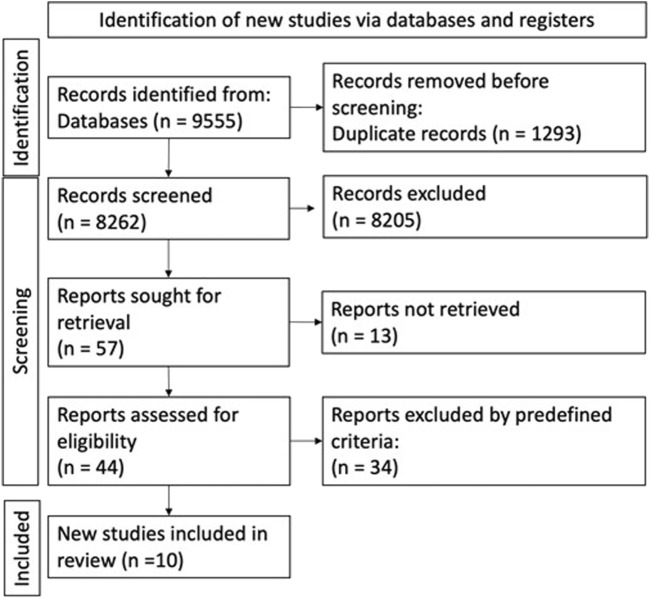
PRISMA flowdiagram of study selection and inclusion.

The goal was to identify randomized controlled examinations (RCTs) therefore comprehensive scans were performed in the databases of PubMed, Embase, Cochrane and Scopus. We defined search keys containing “oesophagus cancer”, “esophagectomy”, “prehabilitation”, “physical examination” and “nutrition” and their variants. The screening process was conducted in two stages. First, two independent reviewers (Cs.P. and A.Cs.) screened all titles and abstracts identified by the search. In the second stage, the full texts of potentially relevant articles were retrieved and assessed for eligibility based on the inclusion and exclusion criteria. EndNote ver. x9.3.3; Alfasoft AB, Göteborg, Sweden) software was used to manage records and identify duplicates. Any disagreements between the reviewers during the screening or data extraction process were resolved through discussion or by consulting a third senior reviewer (A.P.). The screening and selection process is summarized in Figure 1.

The systematic search was conducted in December 2024. We employed a combination of Medical Subject Headings (MeSH) terms and Emtree keywords, along with free-text terms relevant to “esophageal cancer,” “gastric cancer,” “prehabilitation,” and “postoperative complications.” No language restrictions were applied to minimize publication bias. The complete electronic search strings for all databases and the specific search dates are provided in Table 2.

**TABLE 2:** Search strategy.

Search component	Description
Search date	December 2024
Databases	PubMed, embase, cochrane library, scopus
Language restrictions	None (no filters applied for language)
Study design	Randomized controlled trials (RCTs)
Controlled vocabulary	MeSH (PubMed), emtree (embase)
Search filters	Human subjects only
Search strings	(“Esophageal Neoplasms” [MeSH] OR “stomach Neoplasms” [MeSH] OR “esophageal cancer” OR “gastric cancer” OR “upper gastrointestinal cancer”) AND (“Prehabilitation” [All fields] OR “preoperative Care” [MeSH] OR “exercise Therapy” [MeSH] OR “nutritional Support” [MeSH] OR “physical therapy” OR “nutrition”) AND (“postoperative Complications” [MeSH] OR “morbidity” OR “mortality” OR “recovery”) AND (randomized controlled trial[pt] OR “randomized controlled trials as Topic” [MeSH])

### Data extraction

The agreed-upon method was to extract and organize the data into Excel (Office 365, Microsoft, Redmond, WA, United States) spreadsheets for analysis. The following datatypes were extracted: descriptive data for the trials (author, year, type, and number of elements), patients demographics (age, sex, and performance), tumor related data (stage, location), and data related to the therapy received (neoadjuvant regimen, surgical procedure). In addition to this the comparative data on the occurrence of postoperative mortality, clinical or surgical complications, and performance status were also extracted.

### Statistical analysis

The meta-analytic calculations were performed using the STATA statistical software package (StataCorp. 2017. Stata Statistical Software: Release 15. College Station, TX: StataCorp LLC). The working group of the Cochrane Collaborations recommendations was used during the data synthesis. From raw data, the pooled odds ratios (RRs) with 95% confidence intervals (CIs) were calculated for dichotomous variables. In the case of continuous variables, weighted mean differences (WMD) were calculated with 95% confidence intervals. As part of the analysis the random effect model was used with the estimation of DerSimonian and Laird, and the results were displayed on a forest plot. To assess heterogeneity, the Cochrane’s Q and the I2 statistics were used. Statistical significance was achieved in the case of P < 0.05. To account for studies with zero events in one or both arms, a continuity correction of 0.5 was added to each cell of the 2 × 2 table to allow for the calculation of the risk ratio and its variance. Analysis was performed using the random-effects model (DerSimonian and Laird) where significant heterogeneity was observed (I^2^ > 50%), and the common effect model was applied in cases of low heterogeneity.

Sensitivity analyses were performed using the ‘leave-one-out’ method to assess the robustness of our findings and to identify if any single study disproportionately influenced the overall effect size or the heterogeneity.

Trial sequential analysis was performed to assess the necessary number of cases to obtain conclusive evidence in each outcome using the trial sequential analysis tool from Copenhagen Trial Unit (Centre for Clinical Intervention Research, Denmark) [11].

### Risk of bias and certain evidence

Estimating the quality of our investigation, we performed analysis of the article using the Risk of Bias Tool 2 (RoB2) and GRADE approaches.

## Results

During the search process 9,555 articles were identified in the four databases and 8,262 articles remained after removal of duplicates. The selection steps by title, abstract and full text, further narrowed down the number of articles to 1,141 and 57. Finally, after the selection steps, 10 RCTs were included. Cohen’s Kappa was 0.61, which was a substantial agreement between the selection steps of the two authors.

### Characteristics of the studies

All the included international articles used the randomized controlled trial methodology.

For the sake of clarity, the included RCTs are summarized in a table (Table 3).

**TABLE 3:** List of the selected randomized controlled trials used for the meta-analysis.

Author	Title	Year	DOI
Guinan et al.	Effect of preoperative inspiratory muscle training on physical functioning following esophagectomy	2019	10.1093/dote/doy091
Valkenet et al.	Multicentre randomized clinical trial of inspiratory muscle training versus usual care before surgery for oesophageal cancer	2018	10.1002/bjs.10803
Zylstra et al.	Exercise prehabilitation during neoadjuvant chemotherapy may enhance tumour regression in oesophageal cancer: results from a prospective non-randomised trial	2022	10.1136/bjsports-2021-104243
Kong et al.	Effect of perioperative oral nutritional supplementation in malnourished patients who undergo gastrectomy: A prospective randomized trial	2018	10.1016/j.surg.2018.05.017
Liu et al.	Safety, feasibility, and effect of an enhanced nutritional support pathway including extended preoperative and home enteral nutrition in patients undergoing enhanced recovery after esophagectomy: a pilot randomized clinical trial	2020	10.1093/dote/doz030
Loughney et al.	The effect of a pre- and post-operative exercise program versus standard care on physical activity and sedentary behavior of patients with esophageal and gastric cancer undergoing neoadjuvant treatment prior to surgery (the PERIOP-OG trial): a randomized controlled trial	2024	10.1093/dote/doae044
Yamana et al.	Randomized controlled study to evaluate the efficacy of a preoperative respiratory rehabilitation program to prevent postoperative pulmonary complications after esophagectomy	2015	10.1159/000434758
Minnella et al.	Effect of exercise and nutrition prehabilitation on functional capacity in esophagogastric cancer surgery: A randomized clinical trial	2018	10.1001/jamasurg.2018.1645
Allen et al.	Multimodal prehabilitation during neoadjuvant therapy prior to esophagogastric cancer resection: Effect on cardiopulmonary exercise test performance, muscle Mass and quality of Life-A pilot randomized clinical trial	2021	10.1245/s10434-021-11002-0
Bausys et al.	Effect of home-based prehabilitation on postoperative complications after surgery for gastric cancer: randomized clinical trial	2023	10.1093/bjs/znad312

The RCTs involved in the study utilized prehabilitation as an interventional tool at various levels; these, along with the proportion of UGI surgeries and the possible application of ERAS protocol, are summarized in a combined table (Table 1).

### Characteristics of the patients

The mean age was 63.9 ± 5.2 years in the prehabilitation group and 64.2 ± 4.8 years in the control group. The gender distribution was as follows: the prehabilitation group included a total of 122 women and 318 men, while the control group included 118 women and 320 men.

### Characteristics of the tumor and pathological approach

Out of the 10 RCTs, 7 addressed tumor staging. Among these, 6 studies (total number of patients: 523; prehabilitation group: 295; control group: 228) analyzed T(0-)1–4 and N0–3 staging, while one study used the AJCC pathological tumor staging system. Only one study reported T0 stage, including 13 patients in the prehabilitation group and 12 patients in the control group. In the prehabilitation group, T1 stage was recorded in 54 patients, T2 in 53, T3 in 140, and T4 in 27 patients. In the control group, T1 stage was observed in 52 patients, T2 in 40, T3 in 150, and T4 in 27 patients. The AJCC pathological tumor staging system was applied in one study involving a total of 51 patients (prehabilitation group: 26; control group: 25). In the prehabilitation group, 6 patients were classified as stage I, 0 as stage II, and 18 as stage III. In the control group, 5 patients were stage I, 2 were stage II, and 18 were stage III.

### Characteristics of the surgical procedure

The analysis of surgical interventions was challenging due to discrepancies in nomenclature. Following our extensive research on the subject, we found the following: open surgical procedures were examined in four studies, involving a total of 133 patients in the prehabilitation group and 132 patients in the control group. Two articles focused on laparoscopic techniques, with 40 patients undergoing the procedure in each group. VATS (video-assisted thoracoscopic surgery) was reported in two studies, involving 68 patients in the prehabilitation group and 67 in the control group. RATS (robot-assisted thoracic surgery) was performed in 28 patients from the prehabilitation group and 21 from the control group. The transhiatal approach was mentioned in four articles, with 22 patients in the prehabilitation group and 32 in the control group. Transthoracic procedures were described in three studies, including 145 patients who underwent prehabilitation and 141 in the control group. Esophagectomy was reported in two articles, with 21 procedures in the prehabilitation group and 26 in the control group. Partial gastrectomy was described in three studies, with 86 procedures performed in each group. Total gastrectomy was addressed in five articles, involving 45 patients in the prehabilitation group and 44 in the control group. Minimally invasive surgical approaches were discussed in five articles, including a total of 155 patients in the prehabilitation group and 137 in the control group.

### Mortality

**FIGURE 2:**
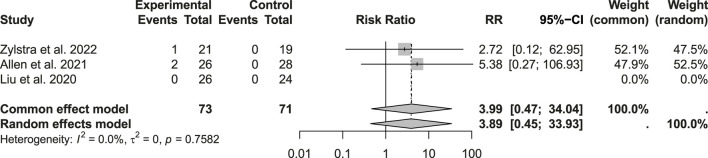
Comparison of mortality within 90 days.

**FIGURE 3:**
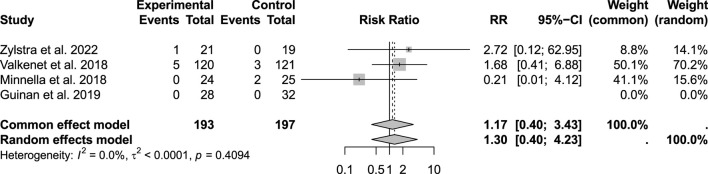
Comparison of in-hospital mortality rates.

“In the included trials, no significant differences were found between the two groups in hospital mortality (p = 0.4094; RR: 1.17; 95% CI: 0.4–3.43; n = 390), 30-day mortality (0 events among 144 patients), or 90-day mortality (p = 0.7582; RR: 3.99; 95% CI: 0.47–34.04; n = 144) (Figures 2, 3). Given that there were no cases of 30-day mortality, this parameter has been omitted from the figure.

### Complication severity analysis

In terms of morbidity, due to the increasingly widespread use of the Clavien-Dindo classification system, we found fewer specific data on the occurrence of individual complications. As a result, we could only identify the wound infection and chyle leak groups, without naming additional categories.

However, the Clavien-Dindo classification establishes a standardized and unified criterion system, which allows for the harmonization of morbidity rates across international studies. The Clavien-Dindo classification defined five complication grades. Grade I includes deviation from normal postoperative course without pharmacological treatment or intervention. Grade II involved complications requiring pharmacological treatment. Grade III referred to complications requiring surgical, endoscopic or radiological intervention with Grade IIIa as a subcategory where intervention happens without anesthesia and Grade IIIb where intervention happens under general anesthesia. Grade IV encompasses cases where life-threatening complications require ICU management, with Grade IVa as a subcategory where a single organ is dysfunctional while Grade IVb includes cases of multi-organ failure. Lastly, Grade V includes cases which resulted in the death of the patients. The grades described here will be used later in the evaluation of the results below [12].

**FIGURE 4:**
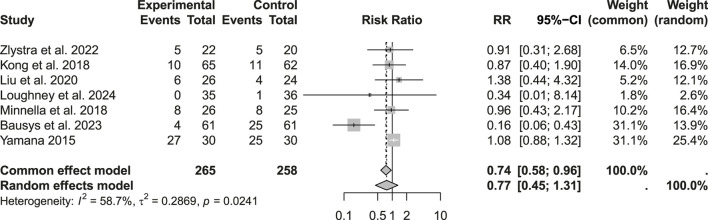
Postoperative morbidity: Clavien-Dindo grade I-II complications.

**FIGURE 5:**
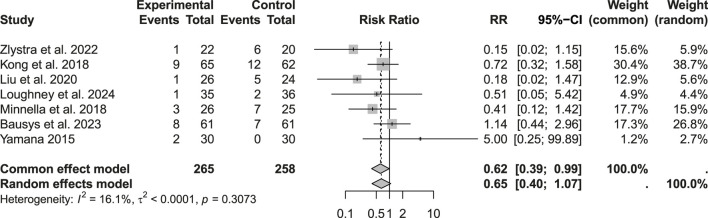
Postoperative morbidity: Clavien-Dindo grade III complications.

**FIGURE 6:**
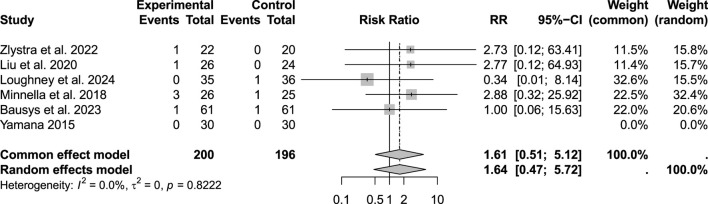
Postoperative morbidity: Clavien-Dindo grade IV complications.

Among the 523 patients analyzed for minor complications (Clavien–Dindo I–II), a reduction was observed in the prehabilitation group (RR: 0.74; 95% CI: 0.58–0.96; p > 0.05). However, considering the heterogeneity and applying a random-effects model, this difference reached only a statistical trend rather than robust significance. Regarding severe complications (Clavien–Dindo Grade III), the analysis of 523 patients showed low heterogeneity, justifying the use of a common effects model. Under this model, prehabilitation was associated with a significant reduction in Grade III complications (RR: 0.62; 95% CI: 0.39–0.99; p < 0.05) (Figures 4–6).

### Surgical complications

**FIGURE 7:**
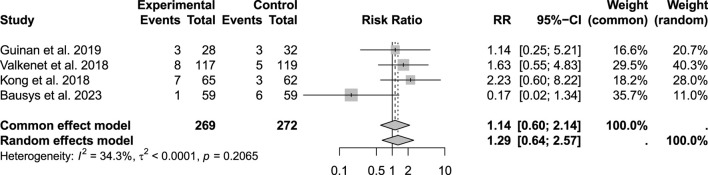
Incidence of wound infection.

**FIGURE 8:**
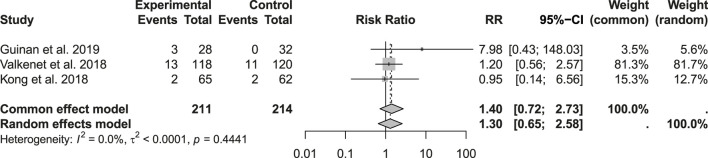
Incidence of postoperative chyle leak.

No significant differences were detected in postoperative surgical complications, with bleeding assessed in 283 patients (p = 0.273; RR: 1.38; 95% CI: 0.45–4.28), wound infection in 541 patients (p = 0.206; RR: 1.14; 95% CI: 0.60–2.14), and chyle leakage in 425 patients (p = 0.44; RR: 1.40; 95% CI: 0.72–2.73) (Figures 7, 8).

**FIGURE 9:**
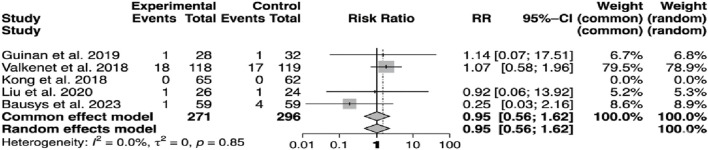
Incidence of anastomotic leakage.

It is crucial to highlight anastomotic leakage, as it remains the most dreaded complication following upper gastrointestinal (UGI) surgery. No significant differences were detected in postoperative surgical complications regarding anastomotic leakage, which was assessed in 567 patients (p = 0.85; RR: 0.95; 95% CI: 0.56–1.62) (Figure 9).

### Other outcomes

**FIGURE 10:**
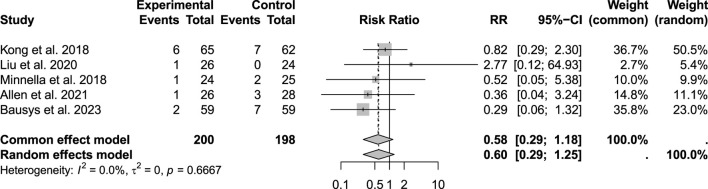
Rates of hospital readmission within the follow-up period.

No significant differences were observed in cardiovascular complications among 543 evaluated patients (p = 0.77; RR: 0.80; 95% CI: 0.51–1.25) or in the risk of readmission among 398 patients (p = 0.66; RR: 0.58; 95% CI: 0.29–1.18) (Figure 10).

The main mortality and morbidity data are summarized in Table 4.

**TABLE 4:** GRADE approach.

Outcome	No. of participants	Relative effect (RR, 95% CI)	p-value	Certainty of evidence	Interpretation
30- day mortality	144	-	-	Very low	No difference detected
90-day mortality	144	RR: 3.99 (0.47–34.04)	0.7582	Very low	No difference detected
In-hospital mortality	390	RR: 1.17 (0.4–3.43)	0.4094	Very low	No difference detected
Minor complications (CD I–II)	523	RR: 0.74 (0.58–0.96)	>0.05 v	Low	Non-significant trend toward reduction
Severe complications (CD-III)	523	RR: 0.62 (0.39–0.99)	<0.05	Moderate	Significantly lower in prehab group (common effects model)
Life-threatening complications (CD-IV)	396	RR: 1.61 (0.51–5.12)	0.82	Very low	No significant difference
Bleeding	283	RR: 1.38 (0.45–4.28)	0.273	Very low	No significant difference
Wound infection	541	RR: 1.14 (0.60–2.14)	0.206	Very low	No significant difference
Chyle leak	425	RR: 1.40 (0.72–2.73)	0.44	Very low	No significant difference
Cardiovascular complications	543	RR: 0.80 (0.51–1.25)	0.77	Very low	No significant difference
Readmission	398	RR: 0.58 (0.29–1.18)	0.66	Very low	No significant difference

## Discussion

While prehabilitation constitutes a core element of the ERAS pathway, Table 1 demonstrates that it is frequently implemented independently of formal ERAS protocols. Unfortunately, the ERAS protocol is still not fully accepted for patients with upper gastrointestinal tumors. As a result, not only is the intraoperative and postoperative protocol not followed—such as early feeding, which has increasing evidence supporting its safety—but prehabilitation is also not implemented in the preoperative period. This is particularly concerning as these patients are often in poor general condition, and prehabilitation could help prepare them physically, nutritionally, and psychologically for surgery. Our goal with this meta-analysis is to highlight the importance and legitimacy of prehabilitation protocol for UGI tumor patients.

Our findings are further supported by a recent 2025 meta-analysis, which examined both randomized clinical trials and cohort studies in patients with upper gastrointestinal cancer. In line with our results, this study demonstrated that prehabilitation significantly promotes postoperative recovery, specifically by shortening the length of hospital stay and improving functional capacity, such as the 6-min walk distance. Notably, it also highlighted a significant reduction in the occurrence of postoperative pneumonia (RR 0.71). Consistent with our analysis, they found no significant differences in major outcomes such as anastomotic leakage, in-hospital mortality, or overall postoperative complications, reinforcing the conclusion that prehabilitation primarily impacts the speed of recovery and the reduction of minor or specific pulmonary events rather than major surgical failures [13].

The present meta-analysis included 10 RCTs, analyzing data from a total of 878 patients who were divided into two groups. The first group consisted of patients who underwent prehabilitation while the second comprised those who were part of the control group.

The complications we examined are best described by the Clavien-Dindo classification [12].

The lower-grade complications are particularly relevant from a quality-of-life perspective, as they often affect patient comfort and recovery. Key examples include atelectasis, pneumonia, wound infections, and infectious diarrhea-conditions that, while not life-threatening, can significantly impact the patient’s wellbeing and prolong recovery time.

Our study observed a notable reduction in minor complications for patients in the prehabilitation group, although this did not reach full statistical significance across all models, suggesting a positive trend toward improved recovery.Our aim was to shed light on the necessity of prehabilitation in patients with upper gastrointestinal tumors.

Examining minor postoperative complications, it was noted that similar findings were reported by Bausys et al. in their study, which included 61 patients in the intervention group and 61 in the control group. Their research analyzed complications such as wound infections, pulmonary complications, anastomotic insufficiency, anaemia requiring blood transfusion, intra-abdominal abscesses, pancreatic complications, postoperative bleeding, cardiovascular and neurological complications, duodenal stump leakage, anastomosis stenosis, and urinary tract infections. An analysis of complication severity revealed that minor complications (Clavien–Dindo grades I–II) occurred significantly less frequently in the prehabilitation group (6.8% compared to 42.4%, P = 0.001; RR 0.16, 0.05–0.43). While initial global assessments suggested no difference, a focused analysis of Grade III complications—which require surgical, endoscopic, or radiological intervention—revealed a significant reduction in the prehabilitation group when using a common effects model, consistent with the low heterogeneity observed for this outcome. (16.9% versus 18.6%, P = 0.810; RR 0.90, 0.41–1.97) [14].

From a mortality perspective, which underscores the importance of prehabilitation for patients in a weakened general condition before surgery, our study found that there was no significant difference between the two groups.

Another study by Allen et al., examined 54 patients (prehabilitation group: n = 26; control group: n = 28) and reported similar findings to ours. According to their results, no deaths occurred within the first 60 days after surgery. In the prehabilitation group, two patients passed away within 90 days of surgical resection, at 66 and 71 days respectively, both due to disease recurrence. There was no significant difference in 3-year cancer-related mortality between the groups [11 patients (50%) in the prehabilitation group vs. 10 patients (43%) in the control group; p = 0.343] [15].

The study of prehabilitation has some limitations. The concept of prehabilitation has many interpretations, encompassing nutritional, physical, and mental prehabilitation. Most studies focused on nutritional and/or physical prehabilitation, while mental prehabilitation received little attention. The randomized controlled trials are not uniform in their outcomes, as demonstrated by the diversity in our meta-analysis. Regarding sample size, the international literature on this topic has not yet shown sufficient interest in this area of medicine, resulting in relatively small patient numbers. Further randomized controlled trials are needed.

In conclusion, this meta-analysis indicates that prehabilitation in patients undergoing surgery for upper gastrointestinal tumors shows a positive trend toward reducing minor postoperative complications (Clavien–Dindo grade I–II), and furthermore, it results in a statistically significant reduction in severe complications (Clavien–Dindo Grade III), although the impact on life-threatening complications (Grade IV) remains non-significant. No significant differences were found in cardiovascular complications or hospital readmission rates either.

These findings support the clinical safety and potential benefit of implementing prehabilitation programs in this patient population.

However, the overall certainty of evidence remains low to very low for most outcomes, highlighting the need for larger, high-quality randomized controlled trials, especially focusing on the role of mental and multimodal prehabilitation, to strengthen the evidence base and standardize clinical protocols in surgical oncology.

## Data Availability

The original contributions presented in the study are included in the article/supplementary material, further inquiries can be directed to the corresponding author.
